# Usefulness of commercially available GPS data-loggers for tracking human movement and exposure to dengue virus

**DOI:** 10.1186/1476-072X-8-68

**Published:** 2009-11-30

**Authors:** Gonzalo M Vazquez-Prokopec, Steven T Stoddard, Valerie Paz-Soldan, Amy C Morrison, John P Elder, Tadeusz J Kochel, Thomas W Scott, Uriel Kitron

**Affiliations:** 1 Department of Environmental Studies, Emory University, Atlanta, GA, USA; 2 Department of Entomology, University of California, Davis, CA, USA; 3 Tulane University, New Orleans, LA, USA; 4 Graduate School of Public Health, San Diego State University, San Diego, CA, USA; 5 US Naval Medical Research Center Detachment (NMRCD), Lima and Iquitos, Peru

## Abstract

**Background:**

Our understanding of the effects of human movement on dengue virus spread remains limited in part due to the lack of precise tools to monitor the time-dependent location of individuals. We determined the utility of a new, commercially available, GPS data-logger for long-term tracking of human movements in Iquitos, Peru. We conducted a series of evaluations focused on GPS device attributes key to reliable use and accuracy. GPS observations from two participants were later compared with semi-structured interview data to assess the usefulness of GPS technology to track individual mobility patterns.

**Results:**

Positional point and line accuracy were 4.4 and 10.3 m, respectively. GPS wearing mode increased spatial point error by 6.9 m. Units were worn on a neck-strap by a carpenter and a moto-taxi driver for 14-16 days. The application of a clustering algorithm (I-cluster) to the raw GPS positional data allowed the identification of locations visited by each participant together with the frequency and duration of each visit. The carpenter moved less and spent more time in more fixed locations than the moto-taxi driver, who visited more locations for a shorter period of time. GPS and participants' interviews concordantly identified 6 common locations, whereas GPS alone identified 4 locations and participants alone identified 10 locations. Most (80%) of the locations identified by participants alone were places reported as visited for less than 30 minutes.

**Conclusion:**

The present study demonstrates the feasibility of a novel, commercially available GPS data-logger for long-term tracking of humans and shows the potential of these units to quantify mobility patterns in relationship with dengue virus transmission risk in a tropical urban environment. Cost, battery life, size, programmability and ease of wear are unprecedented from previously tested units, proving the usefulness of GPS-dataloggers for linking movement of individuals and transmission risk of dengue virus and other infectious agents, particularly in resource-poor settings.

## Introduction

Human movement plays a significant role in the transmission of pathogens, spread of infectious diseases and drug resistance, and emergence of novel pathogens [1-4]. According to its dynamic and complex spatiotemporal nature, human movement can be broadly classified as migratory (i.e., permanent change of residence) or circulatory (i.e., short-term, periodic movements associated with work or leisure) [2,3]. Whereas migration is generally associated with the long-distance spread of disease and the (re)introduction of pathogens into a particular area [2,5], circulation of humans within their activity spaces can be important in the local spread of pathogens and, ultimately, on the fate of disease outbreaks [6,7]. Therefore, knowledge of human spatial behavior is paramount for the development of effective disease surveillance and prevention programs.

Despite its epidemiological relevance, our understanding of the relationship between human movement and pathogen transmission remains limited, in part due both to the lack of precise and cost-effective tools to quantify human spatial behavior and the challenges of data processing [8,9]. Traditional methods (direct observations, diaries and interviews) are plagued with issues of recall, reliability, reproducibility, compliance, behavioral change, and privacy [9-12]. An alternative approach is the use of Global Positioning System (GPS) [12-16] and GPS-cell phone hybrid [17-19] technologies to improve our ability to quantify human movements. However, a variety of factors including cost ($150 for hybrid devices and $450 - $2,000 for custom-built GPS units)[13], size, limited battery life, and dependence on participants to activate units have delayed their adoption in human spatial behavior research and even precluded their use in resource-poor settings.

With a minimum constellation of 24 medium earth-orbiting satellites, the GPS allows the determination of geographic location (i.e., latitude, longitude and altitude), time and velocity with high accuracy anywhere in the world [14,20]. The system works by triangulating the position of GPS receivers with the known locations of 3 or more satellites. GPS satellites emit microwave signals that are used by GPS receivers to estimate positional information. Factors interfering with these signals can affect the spatial accuracy of GPS readings which, without post-processing, is currently around 5-10 m [20]. Currently, GPS receivers not only provide real-time positional information but they also allow recording such information for further mapping and analysis (i.e., data-logging). Research on human activity [11,21] and environmental risk assessment [11,13,14] has been greatly improved by the inclusion of GPS data-loggers. However, applications of this technology to understand the role of human movement on the transmission of vector-borne diseases are lacking [4].

Dengue is a mosquito-borne infection that has reemerged as a major international public health concern over the last four decades [22,23]. Caused by four closely related yet antigenically distinct single-stranded RNA viruses (genus *Flavivirus*, family *Flaviridae*), dengue viruses generally persist in a horizontal *Aedes aegypti*-human transmission cycle [23,24]. Diurnal in their host-biting activity patterns and highly adapted to the human environment, *Ae. aegypti *females seldom disperse beyond 100 m; only a small fraction of mosquitoes was recorded to disperse further than that distance [23,25-29]. Such limited vector dispersal range points to the movement of viremic human hosts within their "home ranges" as a plausible mechanism to explain the rapid spread of dengue in urban environments [30-32]. Although this mechanism was proposed in the early 1980s [30,33] and recently modeled theoretically [4,34], its importance in the dynamics of virus emergence and spread needs to be explored in more detail.

Recent technological advances and consumer demand for location-aware technologies have made commercially available GPS receivers smaller, more accurate and more energy efficient. These developments make GPS much more usable and accessible to researchers who previously would have had to build a custom device. The purpose of this investigation was to determine, in the context of a study on risk of dengue virus infection, the accuracy and effectiveness of commercially available GPS data-loggers for tracking human movements within a resource-poor urban environment.

## Materials and methods

### Study area

This study was performed in the Amazon city of Iquitos (73.2° W, 3.7° S, 120 m above sea level) in the Department of Loreto, northeastern Peru. Because is surrounded on three sides by the Amazon, Nanay, and Itaya Rivers and is accessible only by air or river, Iquitos is considered a geographically isolated city of approximately 400,000 people. Employment in Iquitos is highly informal and dynamic, with 33.4% of the economically active urban population unemployed or informally employed (*Instituto Nacional de Estadística e Informática *of Peru, 2007). The major industries in the area are small commercial enterprises, fishing, oil, lumber, tourism, and to some extent agriculture. Human demography, *Ae. aegypti *entomological indices and dengue epidemiology for Iquitos are described elsewhere [35-37].

### GPS selection

A series of 15 focus group discussions were conducted in January 2008 (with approximately 8-10 participants each, for a total of 144 participants from different age/sex groups) in order to examine whether it would be acceptable for Iquitos residents to wear GPS data-loggers and to explore what types of concerns potential participants would have regarding GPS use [38]. Based on the focus groups' results and the technological needs for tracking of human movements [13], we determined the set of requirements a GPS data-logger unit should meet to effectively track participants' movements in the context of our study: a) data storage capacity and battery life capable of recording at least 3 days of data (to ease mass deployment of units and long-term tracking of individuals); b) durable, water resistant and tamper-proof; c) light weight (i.e., < 100 g); d) unobtrusive design (so that it would not be noticed by others, reducing the chances ofpotential harm from people who might want to steal the units); e) carrying mechanism widely accepted by participants of different ages/sex; f) little to no maintenance required by study participants; and g) low cost. Four commercially available and two custom-made GPS data-loggers were considered as potential candidates (Table 1). Only one of them ("Igot-U GT100", Mobile Action Technology Inc., Taipei, Taiwan) met all of the above criteria and was tested in field experiments.

**Table 1:** Technical specifications of the GPS data-loggers considered as potential candidates for tracking human movements in relationship to dengue virus transmission.

	Model (company)					
	
Features	Igot-U GT100 (Mobile Action)	Photo Trackr (GisTeq)	Trackstick II (Trackstick)	Photo Trackr Lite (GisTeq)	GPS logger v. 2.4, EM-408 (SparkFun)	Custom GPS (Art of Technology)
GPS chipset	SIRF StarIII	SIRF StarIII	ND	SIRF StarIII	SIRF StarIII	Antaris 4
Memory (records)	16,000	16,000	4,000	16,000	Variable (SD card)	58,000
Acquisition at cold/warm start (sec)	35/38	36/33	52/37	36/33	42/38	33/34^1^
Weight including battery (g)	21	68	42	67	28.3 (without battery or case)	75
Dimensions (L/W/H) in mm	44.5/29/13	78/46/23	115/32/19	73/33/31	38/45/23	64/49/16
Water resistant	Yes	No	Yes	Yes	NA^2^	Yes
Programmable	Yes	Yes	Yes	Yes	Yes	Yes
PC connection	USB	USB	USB	USB	SD card	USB
Password protection	Yes	No	No	No	No	No (optional)
Cost (US$)	49	99	150	99	60^3^	> $500^3^

^1 ^Estimate based on alternative architecture; ^2 ^Custom casing required. ^3 ^Unit price depends on volume and features, this price is based on purchase of 100 units;

### GPS field experiments

A suite of experiments was performed to determine the accuracy and effectiveness of the selected GPS data-logger (Igot-U GT100) for tracking human movements in Iquitos. Specifically, we evaluated battery life, spatial accuracy, effect of different sources of interference on spatial accuracy, and feasibility in the determination of an individual's activity space. All experiments were performed between January and April 2008 by UC Davis field technicians. One experiment (Test V) was carried out by two Iquitos residents in September-October 2008, following approval from the University of California, Davis (2007.15244), Naval Medical Research Center Detachment (NMRCD 2007.0007), and Emory University (IRB9162) Institutional Review Boards.

To determine the data collection frequency that maximizes battery life under actual field conditions (Test I) we programmed each GPS model to different collection intervals (i.e., 1 sec, 30 sec, 1 min, 2 min, 2.5 min, 3 min) and distributed them to field technicians who wore units continuously until battery failure. Two different settings that could affect battery life were trialed: the GPS logging positional data only during the day and crepuscule (e.g., from 05:00 to 22:59; "Night-OFF") and the GPS logging positional data at all times (e.g., "Night-ON"). We considered the Night-OFF setting to account for the potential increase of battery life by focusing only on daytime activities that pose the highest risk of exposure to *Ae. aegypti*. A total of 5 repetitions for each collection interval and night logging setting were performed. The time it took each unit to completely discharge was registered.

Spatial accuracy of the selected GPS model was determined by estimating horizontal point (Test II) and linear (Test III) errors as in Elgethun et al. [13]. The horizontal point error represents the average distance from a position registered by a stationary GPS unit to the real position of the unit, whereas the horizontal linear error represents the deviation of the GPS readings taken from moving pedestrians from the real location of their linear paths. The exact geographic location of the known points or paths was derived from a very high (0.6 × 0.6 m pixel size) spatial resolution satellite image (Quickbird, DigitalGlobe, Logmont, CO) georeferenced from high-spatial resolution Iquitos cadastral maps [36]. For the point error, street corners and a park bench were chosen. For the line error several 300 m paths following the external border of urban sidewalks were chosen. A GPS unit was then programmed at a 2-sec interval and left in the identified key location for 2 min (Test II) or taken by a pedestrian while walking the delineated path (Test III). Each experiment was performed by one field technician and repeated three times for each GPS model to account for variations in accuracy due to satellite geometry.

Because GPS data-loggers can be worn differently by study participants (e.g., placed in pockets, purses, back-packs, etc.) we evaluated the effect of such placement locations (which can interfere with the GPS signal) on the horizontal point accuracy of the units (Test IV). Wearing modes tested were a back pocket, a wool purse, a cell phone case and a neck strap. Experiments were performed by one field technician, as in Test II, with the difference that in the first 5 min the device was uncovered and placed in the hand of the technician whereas in the remaining 5 min the device was covered with one of the sources of interference. The field technician remained static on his position at all times during the 10-minute test. Three replicates for each wearing mode were performed to account for variations in accuracy due to satellite geometry (that can translate into variable GPS signal accuracies) and the difference in the horizontal point error between pre- and post-interference was then estimated.

After field testing, the GPS data-loggers were used in a field feasibility test consisting of the assignment of GPS units to two Iquitos residents who, after providing informed consent, wore them for a 15-day period. We selected two men with approximately the same age (45-55 years old), but with different occupations that we expected would translate to different movement patterns. After completion of the GPS trial, participants were asked to recall the places they had visited while wearing the GPS receivers by means of a semi-structured interview, and were given a gift basket with toiletries or food items as exchange for their participation [38]. This test served as a first assessment of the acceptability, compliance, and reliability of the GPS units for mass-deployment and long-term tracking of human mobility patterns.

### Data management and analysis

GPS readings from all experiments were projected from decimal degrees to meters (Universal Transverse Mercator, Zone 18S, WGS1984 datum) and then imported into ArcGIS 9.2 (ESRI, Redlands, CA). For tests II-III, percentage of total points found within a 2.5-, 5-, and 10-meter radius of the real location were estimated as an overall measure of spatial accuracy. Distance from each GPS location to the real location (either a point or a path) was used to estimate the root mean square (RMS) position error of each replicate. RMS represents one of the most used measures of GPS spatial accuracy [20]. For tests II and III, a velocity filter signal correction procedure was performed as in Seto et al. [14]. Briefly, we utilized time and distance between successive locations to filter out those positions that were over a pre-defined (1 m/s) velocity threshold and thus prone to increased error.

In order to estimate the places visited by the two Iquitos residents and the total time spent on them during the 15-day tracking period we implemented the I-cluster algorithm (ICA) developed by Hu and Wang [39]. This data reduction algorithm works by aggregating consecutive GPS readings that are within a spatial (*d*) and temporal (*t*) window, and estimating the total time a participant spent within such spatio-temporal buffer [39]. The algorithm also allows identification of places intermittently visited by applying a threshold time (*tintv*) in between visits. Based on the inherent error of GPS data (e.g., 4-10 m) we determined the following configuration: *d *= 20 m, *t *= 15 min and *tintv *= 30 min, for tracking Iquitos participants. The lot code of the places visited by each participant was assessed by joining the ICA data with georeferenced cadastral maps of the city of Iquitos in ArcGIS 9.2. The shape of the curve of human exposure to an *Ae. aegypti *bite vs the time spent on a location is unknown. In our algorithm we considered 15 min of permanence at a location as an appropriate low-end time (*t*) for an effective human-mosquito contact.

## Results

Four of the six GPS data-loggers considered as potential candidates had a unit cost lower than US$ 100, and presented similar memory capacity, dimensions, signal acquisition times and chipsets (Table 1). However, only one of them ("Igot-U GT100", Mobile Action Technology Inc., Taipei, Taiwan) was additionally water and tamper-proof and allowed for password protection (Table 1). This device was therefore selected for evaluation in actual field experiments in Iquitos. The remaining five GPS models were excluded from further tests.

When collection frequency was set to 1 sec (Test I), the units showed a very low (4.5 h) battery life (Table 2). Increases of collection frequency to 2-3 min allowed collecting up to 2-3 days of data (Table 2). Battery life of the GPS data-loggers was extended at a rate of 17.5 hours per minute increase in the collection frequency. When data collection was disabled between the hours of 23:00 and 05:00, the rate increased to 22.2 hours per minute.

**Table 2:** Effect of data collection frequency and of programming the units to be turned off at night (23:00 to 05:00) on the battery life (Test I) of the Igot-U GT100 GPS data-loggers.

	Average time (in hours) for battery depletion (range)	
	
Time frequency	Night-ON^1^	Night-OFF^1^
1 sec	4.5 (4.4 - 4.5)	ND^2^
30 sec	26.7 (30.3)	34.1 (40.9)
1 min	28.9 (18.4 - 48.1)	42.6 (38.4 - 46.7)
2 min	57.1 (50.8 - 70.3)	60.3 (53.3 - 65.9)
2.5 min	68.5 (61.2 - 73.2)	71.8 (71.7 - 72.0)
3 min	66.3 (51.5 - 76.3)	90.4 (11.3 - 95.0)

^1^GPS units can be programmed to log positions only during the day. Night-ON represents units set to continuously collect positions whereas Night-OFF represented units set to collect positions only during the day and crepuscule (05:00 am to 9:00 pm).

^2^The night option was not tested because of the short battery life of units at this collection frequency.

Table 3 shows the point (Test II) and linear (Test III) horizontal accuracy of the GPS data-loggers. When units were held static in a known position, a point RMS error of 4.4 meters was registered. Most (74.6%) of the data points were located within 5 meters of the known position. The maximum point deviation from the known position was 12.5 m. When the units were taken along a known horizontal linear path, the RMS error increased to 10.3 meters; only 49.4% of the data points were located within 5 meters of the known path. When a velocity filter was applied [14], RMS point error was reduced by only 1.1% whereas RMS line error was reduced by 1.5% (data not shown).

**Table 3:** Point (Test II) and linear (Test III) spatial accuracy of the Igot-U GT100 GPS data-loggers.

						Percentage of locations within		
						
Test*	Number of locations	Mean	SD	Max	RMS**	2.5 m	5.0 m	10.0 m
*Test II*	1,535	3.9	2.1	12.5	4.4	27.3	74.6	98.2
*Test III*	1,669	7.6	6.9	23.2	10.3	28.3	49.4	69.2

* Represents the horizontal point error (Test II) and the horizontal linear error (Test III) of each GPS model. ** RMS, root mean square error.

**Figure 1:**
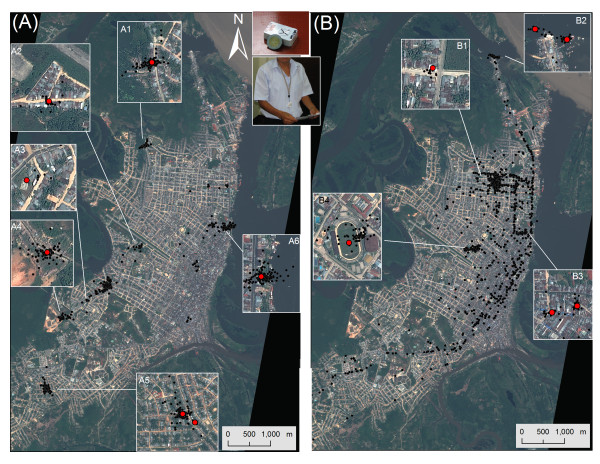
**Locations registered by Igot-U GPS data-loggers assigned to a carpenter (A) or a moto-taxi driver (B) for 14-16 days (black dots)**. Insets zoom to the locations identified by the I-clustering algorithm (red dot) as most frequently visited by each participant. Pictures show the size of the Igot-U GPS data-logger and the neck-strap wearing mode. Location of participants' houses is not presented to preserve their identity.

Among the different potential sources of GPS signal interference tested (Test IV), back pockets proved to increase the RMS point error the most (Table 4). The difference in RMS errors before and after placing the GPS data-loggers on each source of interference decreased from 6.9, 4.0, 3.6 and 0.0 meters for the back pocket, cell phone case, wool purse and neck strap, respectively (Table 4). Neck straps (Figure 1) proved not only to produce the least signal interference but, according to our focus group discussions [38], also to be the most versatile and preferred wearing mode for our study population.

**Table 4:** Effect of different sources of interference (Test IV) on the spatial accuracy (measured as the root mean squared error, RMS) of the Igot-U GT100 GPS data-loggers.

Source of interference	Number of locations	RMS before^1^	RMS after^2^	ΔRMS
Neck strap	784	6.2	6.2	0.0
Wool Purse	825	5.1	8.7	3.6
Cell phone case	822	4.4	8.4	4.0
Back Pocket	831	2.6	9.5	6.9

^1 ^Before applying the source of interference (i.e., the GPS was held in the hand of a field technician in an open area with clear view of the sky).

^2 ^After introducing the source of interference. The technician remained static on the same position for the duration of the test.

In the field feasibility test a carpenter (participant A: male, 55 years old) and a moto-taxi driver (Participant B: male, 47 years old) consented to wear an I-gotU GPS data-logger at all times for 15 days. Participants were asked to wear the units on a neck strap to reduce signal interference. GPS data-loggers were programmed to collect data points at all times every 2.5 min. Every 3 days units were retrieved and exchanged for a second one, to allow for continuous recording of participants' movements. During the first 3-day period the GPS units from both participants were retrieved without any data, probably because they were mistakenly delivered with the power button enabled, and were therefore turned off while handled by the participants. Given this undesired data-collection issue the tracking period was extended 4 more days. When only days with GPS data were considered, participant A and B, reported use of the units for 100% (14/14 days) and 93.8% (15/16 days) of the time, respectively. Participant B reported forgetting the GPS at home on one day, and not using it from 7:00 am to 6:00 pm on that day. Besides forgetting the GPS unit on one day, participants did not report any other complaint or limitation during the tracking period.

Figure 1 shows the GPS readings from each participant together with the locations identified by the I-cluster algorithm. Locations visited for a significant amount of time were identified by the presence of a "cloud" of data points. The integration of I-cluster derived GPS locations and semi-structured interview data shows that, overall, participant A seemed to move less and to spend more time in more fixed locations than participant B (Figure 1; Table 5). Both residential and non-residential locations were visited by study participants but in a different proportion:44% (4/9) of locations visited by Participant A were residential whereas such value was 18% (2/11) for Participant B (Table 5). Additionally, the I-cluster algorithm allowed estimating the total time each participant spent at each location and the frequency of visits to that location while tracked (Table 5), two key parameters describing human spatial behavior.

**Table 5:** Frequency and duration of permanence in different locations over 14-16 days registered by GPS readings (using the I-cluster algorithm) and semi-structured interviews administered to two randomly selected participants from the city of Iquitos, Peru.

		GPS readings			Semi-structured interview	
			
Site code	Type	Frequency (times visited)	Duration of each visit (hours)	Total time (hours)	Frequency (times a week)	Duration of each visit (hours)
A1	House	2	6	12.1	2	12.00
A2	House	1	3	3	2	15.00
A3	School	1	0.5	0.5	2	2.00
A4	House	3	1.8	5.4	ND^1^	ND
A5	House	3	6	18	ND	ND
A6	Port	8	1.1	8.5	5	2.00
A7	Market	ND	ND	ND	1	0.33
A8	Store	ND	ND	ND	7	0.33
A9	Market	ND	ND	ND	7	0.17
B1	House	1	0.5	0.5	ND	ND
B2	Market	1	2	2	2	1.00
B3	Market	6	0.25	1.6	1	0.33
B4	Stadium	3	1.8	5.5	ND	ND
B5	Market	ND	ND	ND	4	0.33
B6	Market	ND	ND	ND	4	0.33
B7	Park	ND	ND	ND	1	0.25
B8	Park	ND	ND	ND	2	0.33
B9	Hospital	ND	ND	ND	1	0.42
B10	Church	ND	ND	ND	1	1.00
B11	House	ND	ND	ND	1	8.00

^1^ND: represents sites not identified by the respective method.

For information on site codes refer to Figure 1.

The concordance between GPS readings and participants' recall about frequency and duration of each visit was low and variable among sites (Table 5). GPS and participants' interviews concordantly identified 6 common locations, whereas GPS alone identified 4 locations and participants alone identified 10 locations. Most (80%) of the locations identified by participants alone were places reported as visited for less than 30 minutes. Two places identified by participants alone and visited for 1 hour or more (B10, B11) were not identified by GPS most likely because they were located in the house next to the participants' houses and therefore could not be disentangled from the point cloud neither by the I-cluster algorithm nor visually (data not shown).

## Discussion

The spatio-temporal dimension of dengue virus transmission risk is determined by the interplay of multiple factors such as the level of herd immunity in the human population; virulence characteristics of the circulating viral strain; temperature and rainfall; survival, abundance, dispersal and blood feeding behavior of female *Ae. aegypti*; and human density and age structure [30,33]. The effect of many of such factors on dengue virus transmission risk can be modulated by the movement of susceptible individuals into locations where they can be infected, or of infective individuals into locations where virus can be transmitted to susceptible people [30,33]. Therefore, contact rates between humans and mosquitoes are not random [40], but highly dependent on the locations each person visits during his/her daily activity routine. Considering the limited (< 300 m) *Ae. aegypti *dispersal distance [23,25-29,36] and that dengue virus is efficiently transmitted even at very low *Ae. aegypti *population densities [30], it is expected that the measurement of risk factors across the locations most frequently visited by a person may increase our ability to better predict individual risk to dengue virus infection and better define the spatial dimensions for surveillance and control. The present study demonstrates the feasibility of a novel, commercially available GPS data-logger for long-term tracking of humans and shows the potential of these units to quantify mobility patterns in relationship with dengue virus transmission risk in a tropical urban environment. This approach for linking movement of individuals (or permanence in particular locations) with disease transmission risk may prove useful for the research of other infectious diseases for which mobility is key for understanding pathogen emergence, persistence and spread [4].

Dengue vector control failures are partly due to our deficiencies in understanding relationships among available interventions, virus transmission dynamics and human behavior [41]. A classic approach to suppress dengue transmission during the early stages of an epidemic consists on the targeted implementation of vector control actions (e.g., source reduction and/or insecticide sprays) within a buffer distance (e.g., 50-100 m) of a confirmed case's residence [42]. Such approach may be prone to failure if transmission occurred in places other than the residence. Hence, assessing the most likely place of transmission is vital for the success of vector control campaigns, particularly during the early stages of a dengue epidemic. Although vector control agencies can deem GPS technology as an impractical tool to monitor retrospectively where a suspected dengue case has spent his/her daytime during the previous 15 days, the implementation of such technology can help them better design survey instruments geared to determine the most likely place(s) of exposure to infective mosquito bites.

Several features made the GPS data-loggers tested in the present study suitable for tracking human movements, particularly in resource-poor environments. The GPS cost, battery life, size, weight and shape, ease of programming and deployment are unprecedented compared with previously tested units [13-16,18,19,41]. Their cost made them affordable for large-scale distribution; their small size and light weight helped in the high acceptance of the devices by study participants, both in terms of their all-day wearability, as well as being discrete enough to be unnoticeable to others [38]. Password protection and a special adaptor for downloading data allows for protection of participant confidentiality. Protection of personal information was identified as a significant concern by Iquitos residents participating in focus groups [38] and also represents a major requirement for any study involving the collection of sensitive positional information. Furthermore, the GPS data-loggers we used can be easily deployed in resource-poor settings, because they require minimum involvement by study participants (only wearing the units when leaving the house, periodic visits by research assistants and a brief questionnaire); the consideration of such factors prevented us to consider other popular location-aware technologies. GPS hybrid cell phones were not considered because not only are more expensive (US$150 plus the cost in local calls made by each participant) but are also more cumbersome to use (particularly by older participants), are difficult to encrypt to prevent undesired access of the positional data, are more prone to be stolen and can be subject to difficulties at the end of the tracking period (by having participants willing to keep the units because of their nicer design and features). By performing focus groups discussions with Iquitos residents and taking into account their potential concerns with regard to the implementation of GPS technology to track their movements [38] we not only were able to determine the set of features a GPS unit needs to be used in resource-poor settings but also were able to adapt wearing modes and GPS exchange schemes to increase the acceptance and compliance of the tested units (evidenced by the high frequency of use and lack of complaints by the study participants). The use of a particular GPS model in this study did not mean we are endorsing a particular brand. Rather a particular set of features were needed for accurately tracking human movements in resource-poor settings. We believe that the rapid advance in GPS technology will bring in the future units capable of performing equally or even better than the ones tested in this work.

All GPS units show a trade-off between frequency of data collection and battery life, which ultimately should be addressed by determining when and why a very detailed (e.g., second-by-second) tracking of participant movements is needed. Research on human spatial behavior shows that the frequency of newly-visited sites decays as a function of time [9,43], suggesting that GPS data from more than one day would be needed to determine the locations where regular activities occur. On one hand, a small collection interval (e.g., every 1-5 sec) will provide detailed information about the roads and paths taken to connect each location visited, but will also make long term tracking of individuals more difficult. On the other hand, a large collection interval (e.g., 2.5 min) increases battery life but, as observed in our study, increases the risk of missing short-term visits. In our study, we focused on the locations visited by participants rather than the paths followed by them to reach such locations; increasing the collection interval to 2.5 min not only allowed us to detect such locations but also resulted in an enormous logistical advantage by allowing the coordinated deployment and retrieval of units over a longer period of time. Furthermore, battery life could have been increased by distributing the GPS units with an AC battery charger. However, issues of acceptability and compliance by the residents need to be assessed before full implementation of such strategy.

The degradation of GPS radio signals due to bad satellite geometry, signal deflection between the satellite and the receiver and extremes in upper atmospheric conditions are the major sources of GPS positional inaccuracy [20]. In Iquitos, buildings rarely have more than two stories and streets are wide, eliminating the detrimental effect that "urban canyons" can have on GPS accuracy. In contrast to previous assessments [13], we estimated positional accuracy in the present work without differential correction and by replicating each experiment in different days and sectors of the city. This allowed us to better capture variations in GPS positional accuracy due to the different sources of error. The horizontal point errors registered with the GPS data-loggers were only 3 to 7 meters greater than the ones registered with more expensive custom-designed units for which a differential correction of the signal was necessary [12,14]. Moreover, the application of a velocity filter did not increase significantly the point and line accuracy. This may be due to the SiRF StarIII chipset's ability to acquire and maintain a signal lock in urban or densely covered forest environments, and its faster time to lock onto the satellite signals and determine the initial position. In addition, the negative effect of clothing on GPS positional accuracy was reduced by asking participants to wear the units attached to neck straps. Participants showed high acceptance of this feature and did not report any problems during the tracking period. Compared to custom-designed GPS vests [13], GPS data-loggers have not only proved to be similarly accurate, but also to be more convenient for tracking people without affecting their normal behavior, especially given their small size and versatile wearing modes.

Although performed with only two volunteers, our field test showed interesting and contrasting results in the observed movement patterns of study participants. Differences in the number and proportion of residential sites visited and frequency and duration of each visit were most likely the result of participants' occupations. Participant B, a moto-taxi driver, moved more and visited more public (e.g., markets, parks, port, hospital) than residential locations for short (less than 30 min) periods of time. Such spatial behavior differs from Participant A, who tended to visit more residential locations and spend more time in those sites. In Iquitos both residential and public locations have the potential of supporting *Ae. aegypti *populations [37,44]. The relative contribution of each type of location to the transmission of dengue virus transmission has never been assessed.

In our study, the signal collection frequency of 2.5 minutes precluded the detection of many sites that were only rarely visited (once and by less than 30 minutes during a 15 day period) by study participants. Additionally, several sites reported as visited and located close (< 20 m) to each other were not detected by the clustering algorithm because their position could not be untangled from the data cloud. Although missing such locations can represent a disadvantage for the proper implementation of GPS technology we also consider that, in the context of our dengue study, it won't represent a serious problem. By searching for mosquitoes within 20-30 m of each GPS identified location (within *Ae. aegypti *flight dispersal and spatial clustering [36] distance) we not only would be able to account for the lack of fine-scale resolution of GPS technology but also would increase our chances of finding infected mosquito females responsible for virus transmission. Furthermore, because our research aims to identify the locations where effective *Ae. aegypti*-human contact and virus transmission may have occurred, we do not consider missing some rarely or briefly visited (< 30 min in a 15-day period) sites to be a serious handicap for our ability to assess the activity patterns of study participants in our study. To acquire a bloodmeal *Ae. aegypti *females need first to be attracted to a host and then effectively attack and feed from such host. Although there is no empiric evidence describing the time it takes *Ae. aegypti *females to perform this complex repertoire of behavioral and physiological traits, we expect it to be dependent on the time a person spends on a location. By excluding locations rarely visited during the tracking period our study will allow focusing on the places in which participants are most likely to be exposed by mosquito bites. This exclusion of sites may prove difficult for other diseases in which there is not a time-dependence in the exposure to the source of pathogens.

Beyond spatial and temporal accuracy, one of the main challenges in the implementation of GPS data-loggers is that the large amounts of positional data they collect needs to be processed in order to determine the locations and times where virus exposure may have occurred. By tracking participants' movements for a longer period of time (i.e., more than a week), frequented locations were easily identifiable by the accumulation of redundant data (observed by the presence of data "clouds" around such locations). Such characteristics of human spatial behavior can be exploited by GIS-based clustering algorithms like the one employed in this study [39]. Such algorithm effectively allowed to determine which locations were significant (e.g., frequently visited by study participants), and which could be ignored. In the context of our dengue study, the identification of locations regularly visited by the participants together with the time spent on each location will be further used to develop spatially explicit dengue transmission models [7]. Under this analytical framework a participant's individual risk of exposure to dengue infection would be directly proportional to the number of sites (including his own house) he visits during regular daytime activities, the time spent on each site and the local abundance of infected *Ae. aegypti *females on each site [4]. This conceptual model can be represented as a bipartite network in with nodes representing individuals are linked to nodes representing the locations visited by them [6], with the weight of the links representing the time spent on each location times the local abundance of *Ae. aegypti *females. Most research on bipartite contact networks have relied on individual-based model-derived contacts (see [6] for an example). By adopting GPS technology we expect to empirically derive a contact network that can later be incorporated in dynamic mathematical models of dengue transmission to account for complex patterns of human interaction and movement.

The finding of discordant results between GPS and questionnaires is not uncommon [12,14,15,19,45,46]. Questionnaire accuracy can be affected by conscious omission by the interviewee, interviewer error (e.g. inability to prompt recall), and recall bias (the further in time participants are asked the lower their accuracy) [9,10], whereas accuracy of GPS technology is affected by the point and line spatial errors, the signal collection frequency and the ability to transform data clouds into visited locations. Our field study showed differences between GPS and semi-structured interviews in the time spent on each location as well as in the number of locations identified by each method. However, the low number of participants included in this study prevented us to perform any thorough statistical comparison between methods. We are currently performing a trial involving 120 participants to compare the accuracy of a semi-structured interview instrument with GPS observations with the aim of developing an improved toolbox geared to help overcome problems with recall and reliability inherent to studies of human movement and dengue virus transmission.

## Competing interests

Author Tadeusz J. Kochel is a U.S. military service member. This work was prepared as part of his official duties. Title 17 U.S.C. §105 provides that 'Copyright protection under this title is not available for any work of the United States Government'. Title 17 U.S.C. §101 defines a U.S. Government work as a work prepared by a military service members or employees of the U.S. Government as part of those person's official duties.

None of the authors has a financial or personal conflict of interest related to this study. The corresponding author had full access to all data in the study and final responsibility for the decision to submit this publication.

## Authors' contributions

All authors contributed equally in the conception and design of the study. GVP and STS helped collect the data, carried out the analysis and drafted the article. All authors read and approved the final manuscript.
